# Comparison of the Nutritional Quality of Branded and Private-Label Food Products Sold in Italy: Focus on the Cereal-Based Products Collected From the Food Labeling of Italian Products Study

**DOI:** 10.3389/fnut.2021.660766

**Published:** 2021-06-09

**Authors:** Donato Angelino, Cristian Del Bo', Nicoletta Pellegrini, Daniela Martini, SINU Young Working Group

**Affiliations:** ^1^Faculty of Bioscience and Technology for Food, Agriculture and Environment, University of Teramo, Teramo, Italy; ^2^Department of Food, Environmental and Nutritional Sciences, Università degli Studi di Milano, Milan, Italy; ^3^Department of Agricultural, Food, Environmental and Animal Sciences, University of Udine, Udine, Italy

**Keywords:** cereal, food labeling, brand, private label, nutrition and health claims, nutrition declaration

## Abstract

The packaged foods sold in food stores may be “private-label” products (PL), when branded by the supermarket, and “branded” products (BR). PL products are generally cheaper than the BR counterparts, and this can be perceived as a sign of general low quality by consumers, when items are compared with their branded counterparts. Thus, the aim of the present study was to compare the nutrient content of BR and PL cereal-based foods, by evaluating the nutritional declaration reported on the food pack of products on the home-shopping website of major retailers present on the Italian market. A total of 3,775 items (~58% BR and ~42% PL), collected in the period from July 2018 to March 2019 and updated in March 2020, were included in the final analysis. Data were analyzed by means of the Mann–Whitney nonparametric test for two independent samples for differences between BR and PL categories and types. Overall, BR products showed higher contents of total and saturates than PL items. When products were grouped for categories and types, items only differed for the content of total fats, saturates, total carbohydrates, proteins, and salt. No differences were instead found for energy and sugar contents among any of the categories. However, we did not find any consistency in the direction of results. These results could be useful for future education activities aimed to help consumers in making informed food choices.

## Introduction

Food labels are one of the main tools used by industries to deliver information about their products. Among the different information reported on the food pack, the brand name and/or logo is certainly one of the main aspects that majorly attracts the customer's interest during shopping time, linking the product to positive feelings, and in turn may influence purchasing behavior and consumption (1). Regarding the brand, in the last decades, two classes of products have been proposed to the customers: “private-label” (PL) and “branded products” (BR). The first class describes packaged food items of all food categories generally produced by small-medium food companies (i.e., copacker) but branded by the supermarket and sold exclusively in the supermarkets' own stores. These products, also called “own label,” are considered a competitive alternative to the so-called “branded products,” which are produced by national and international food manufacturers and labeled with their own brand and distributed to the general trade (2).

PL products are constantly gaining market shares despite some signs of declining in the last years (3). However, they are not equally distributed around the world, being more diffused in Europe and less penetrated in the Asia Pacific (4). Large variability in terms of penetration also exists for the different categories of food products: for some categories that do need a higher level of confidence (e.g., baby food), consumers show indeed a higher attitude for BR compared with PL (5). In Italy, the share value for PL products has been reported to be ~20%, with some more performing categories, such as eggs and frozen vegetables, and others with a lower value share such as biscuits, pasta, and snacks (6). The balance between BR and PL in the market represents a critical task. In fact, on the one hand, branded food companies drive traffic and variety of products and are more engaged in the innovation processes, while PL products are essential for increasing the retailer's value image and profitability and, at the same time, can be used by consumers as a mean to save money (4). PL products were indeed initially developed to emulate traditional national brands and based their success on price since they have always been considered a cheaper choice than BR. In fact, an Australian study compared the cost of BR food products with their PL equivalents across a range of food categories, reporting a 44% cost saving by purchasing PL over BR products (7). It is undeniable that, mostly in the past years, the association between the absence of a known brand and low price led to the perception of PL as products with lower nutritional quality compared with BR items (8).

Few studies have been performed investigating the potential difference between PL and BR products in terms of nutritional quality, focusing on specific food components such as sodium (9) or considering a wide range on nutrients (10, 11). However, to the best of our knowledge, no studies compared the nutritional quality of BR and PL on the Italian market, also considering the prevalence of nutrition and health claims (NHC).

Based on these premises, the aim of the present study was to compare energy and nutrient contents of BR and PL cereal-based foods currently sold in Italy, by evaluating the nutritional declaration reported on the food pack. This work is part of the Food Labeling of Italian Products (FLIP), conceived by the Working Group SINU (Italian Society of Human Nutrition) Young with the purpose of systematically evaluating the nutritional quality of the different food categories sold on the Italian market (12–17) and of elucidating whether specific information related to the food pack can be considered markers of overall nutritional quality.

## Materials and Methods

### Product Selection and Data Collection

The online search for information was conducted on the home-shopping website of the major retailers present on the Italian market (Auchan, Bennet, Carrefour, Conad, Coop Italia, Crai, Despar, Esselunga, Il Gigante, Iper, Pam Panorama, Selex, Sidis). We included all the prepacked cereal-based foods for which, as stated in the Council Regulation (EC) no. 1169/2011 (18), mandatory food information shall appear directly on the package or on a label attached thereto.

The exclusion criteria for product selection were as follows: (i) not prepacked, (ii) incomplete images of all the sides of the pack, (iii) unclear images of nutrition declaration or list of ingredients, and (iv) products marked as “product currently unavailable” in all the online stores which were selected throughout the data collection period. The online research was performed from July 2018 to March 2019 and updated on March 2020.

### Data Collection

Data from the complete images of all the sides of the pack were collected for all the selected products. As previously described, the following qualitative–quantitative and specifically regulated (mandatory) information was retrieved for each food item: company name, brand name, descriptive name, energy (kcal/100 g), total fat (g/100 g), saturated fatty acids (SFA, g/100 g), total carbohydrates (g/100 g), sugars (g/100 g), protein (g/100 g), and salt (g/100 g). Moreover, the number of nutrition claims (NC) and of health claims (HC), as listed in the Council Regulation (EC) No. 1924/2006 (19), was collected.

Data were extracted once but the accuracy of the extracted data was double-checked by two researchers (CDB, DA), and inaccuracies were resolved through secondary extractions made by a third researcher (DM).

A dataset was created with all the collected data and items were subgrouped for specific comparisons by considering the descriptive name reported on the pack and the brand. Based on the brand, the food items were classified into (i) private-label foods for products branded by the supermarket and (ii) branded foods for food items produced by food manufacturers and labeled with their own brand. Based on the descriptive name, food items were further divided into the following categories and into related types, as previously reported (12, 13, 15–17): (i) breakfast cereals (cereal bars, muesli, flakes, bran cereals, puffed cereals, and others), (ii) biscuits (tea cookies, shortbread biscuits, cream-filled wafer, covered and/or sandwich cookies, Italian traditional biscuits, and other biscuits), (iii) sweet snacks and cakes (cream-filled sponge cake, plain or cream/jam-filled croissant or “pain au chocolat,” yogurt plumcake and muffin, sponge cake, cream/jam-filled shortbread cake, cream-filled and/or covered chilled snack), (iv) bread (loaf, rolls and sliced bread), (v) bread substitutes (rusks, wraps, rice and corn cakes, crackers, breadsticks, “croutons, bruschetta, and frisella bread,” and taralli), (vi) fresh pasta (semolina, egg, stuffed pasta), and (vii) dried pasta (semolina, egg, stuffed, special pasta).

### Statistical Analysis

Statistical analysis was carried out using IBM SPSS Statistics® (Version 25.0, IBM Corp., Chicago, IL, USA) and performed at *p* < 0.05 of significance level. The normality of data distribution was firstly verified through the Kolmogorov–Smirnov test and rejected. Therefore, variables were expressed as median and interquartile range. Data of energy and nutrient contents per 100 g of products for each item were analyzed by means of the Mann–Whitney non-parametric test for two independent samples for differences between BR and PL categories and types. Comparisons among product types of each category were shown graphically using Origin software (OriginPro 2019, OriginLab Corp., Northampton, MA).

## Results

### Number and Characteristics of Retrieved Food Items

Table 1 reports the number and type of retrieved items, with a total of 3,775 items included in the final evaluation, among which ~58% were BR and ~42% were PL products, respectively. The most numerous food categories were bread substitutes with over 1,000 items and biscuits with 814 items; the least numerous categories were bread with 339 items and fresh pasta with 269 items. For all categories, the number of BR items was higher than that of PL label ones, up to two-thirds of the total mostly for biscuits and dried pasta.

**Table 1:** Number and types of private-label (PL) and branded (BR) items.

**Category**	**Brand**	**Number of items**	
Breakfast cereals (*n* = 370)	PL	176	Cereal bars (*n* = 15), muesli (*n* = 26), flakes (*n* = 81), bran cereals (*n* = 6), puffed cereals (*n* = 16), and others (*n* = 32)
	BR	194	Cereal bars (*n* = 62), muesli (*n* = 28), flakes (*n* = 48), bran cereals (*n* = 8), puffed cereals (*n* = 13), and others (*n* = 35)
Biscuits (*n* = 814)	PL	310	Tea cookies (*n* = 69), shortbread biscuits (*n* = 173), cream-filled wafer (*n* = 32), covered and/or sandwich cookies (*n* = 14), Italian traditional biscuits (*n* = 17), and others (*n* = 5)
	BR	504	Tea cookies (*n* = 184), shortbread biscuits (*n* = 184), cream-filled wafer (*n* = 44), covered and/or sandwich cookies (*n* = 64), Italian traditional biscuits (*n* = 17), and others (*n* = 11)
Sweet snacks (*n* = 476)	PL	227	Cream-filled sponge cake (*n* = 11), plain or cream/jam-filled croissant or “pain au chocolat” (*n* = 76), yogurt plumcake and muffin (*n* = 83), sponge cake (*n* = 36), cream/jam-filled shortbread cake (*n* = 21), cream-filled and/or covered chilled snack (*n* = 0)
	BR	249	Cream-filled sponge cake (*n* = 40), plain or cream/jam-filled croissant or “pain au chocolat” (*n* = 74), yogurt plumcake and muffin (*n* = 66), sponge cake (*n* = 53), cream/jam-filled shortbread cake (*n* = 10), cream-filled and/or covered chilled snack (*n* = 6)
Bread (*n* = 339)	PL	141	Loaf (*n* = 34), rolls (*n* = 26), and sliced bread (*n* = 81)
	BR	198	Loaf (*n* = 67), rolls (*n* = 44), and sliced bread (*n* = 87)
Bread substitutes (*n* = 1,020)	PL	424	Rusks (*n* = 48), wraps (*n* = 65), rice and corn cakes (*n* = 60), crackers (*n* = 93), breadsticks (*n* = 71), “croutons, bruschetta, and frisella bread” (*n* = 49), and taralli (*n* = 38)
	BR	596	Rusks (*n* = 69), wraps (*n* = 81), rice and corn cakes (*n* = 114), crackers (*n* = 93), breadsticks (*n* = 126), “croutons, bruschetta, and frisella bread” (*n* = 51), and taralli (*n* = 62)
Fresh pasta (*n* = 269)	PL	131	Semolina (*n* = 14), egg (*n* = 21), stuffed pasta (*n* = 96)
	BR	138	Semolina (*n* = 2), egg (*n* = 24), stuffed pasta (*n* = 112)
Dried pasta (*n* = 487)	PL	173	Semolina (*n* = 68), egg (*n* = 71), stuffed (*n* = 1), special pasta (*n* = 33)
	BR	314	Semolina (*n* = 89), egg (*n* = 135), stuffed (*n* = 4), special pasta (*n* = 86)
Total	PL	1,582	
	BR	2,193	

Concerning NHC, the number of products with at least one nutrition claim or health claim was higher among BR products compared with PL items, except for fresh pasta in which PL prevailed on BR (*n* = 2 and 1, respectively) (Supplementary Table 1).

### Nutritional Quality of Branded and Private-Label Food Categories and Types

The values of energy, macronutrients, and salt content of branded and private-label food categories are summarized in Table 2. Considering all the 3,775 items, BR and PL items statistically differed only for contents of total fats and SFA that were higher in the former [total fat: 10.1 (4.2–18.0) vs. 9.6 (4.1–17.0) g/100 g, *p* = 0.025; SFA: 2.0 (0.9–5.2) vs. 1.9 (0.1–4.8) g/100 g, *p* = 0.025, in BR and PL, respectively]. As regards the seven categories, overall, no differences were found for energy content in any of the considered categories, while only small differences were observed in specific nutrient content for some products. More specifically, higher total fat and SFA contents were observed in BR compared with PL products for breakfast cereals [total fat: 7.4 (2.9–15.0) vs. 3.9 (1.7–7.5) g/100 g, *p* < 0.001; SFA: 1.9 (0.7–4.1) vs. 1.0 (0.4–3.0) g/100 g, *p* < 0.001, in BR and PL, respectively], sweet snacks [total fat: 19.0 (16.0–22.0) vs. 17.1 (15.0–21.0) g/100 g, *p* = 0.046; SFA: 7.6 (4.1–10.4) vs. 6.5 (3.7–9.4) g/100 g, *p* = 0.006], and dried pasta [total fat: 2.8 (1.7–4.2) vs. 2.0 (1.4–3.8) g/100 g, *p* < 0.001; SFA: 0.8 (0.4–1.3) vs. 0.5 (0.3–1.2) g/100 g, *p* = 0.047, in BR and PL, respectively], while BR and PL fresh pasta differed for total fat [8.0 (5.5–10.0) vs. 6.5 (3.8–8.4) g/100 g, *p* = 0.002] but not for SFA. Regarding total carbohydrates, a lower content was observed in BR products than PL counterparts for breakfast cereals [65.2 (57.0–75.0) vs. 76.0 (64.8–81.0) g/100 g, *p* < 0.001, for BR and PL, respectively], sweet snacks [52.0 (49.0–56.6) vs. 54.0 (51.0–58.0) g/100 g, *p* < 0.001], and dried pasta [68.0 (66.0–71.0) vs. 68.0 (67.0–71.5) g/100 g, *p* = 0.010, for BR and PL, respectively], while no differences were found for sugar content in any of the food category under study. Contrasting results were observed for protein, with a higher content in BR breakfast cereals [8.6 (7.3–11.0) vs. 8.0 (7.0–9.5) g/100 g, *p* = 0.027] and dried pasta [14.0 (12.5–15.0) vs. 13.0 (12.0–14.0) g/100 g, *p* < 0.001, for BR and PL, respectively] compared with the PL counterparts, while a lower protein content in BR products compared with PL was found for fresh pasta [9.9 (8.7–11.0) vs. 11.0 (9.3–13.0) g/100 g, *p* < 0.001]. Similarly, a higher salt content was observed in BR compared with PL biscuits [0.6 (0.4–0.8) vs. 0.5 (0.3–0.7) g/100 g, *p* = 0.004], fresh pasta [1.2 (0.7–1.4) vs. 0.9 (0.3–1.3) g/100 g, *p* = 0.004], and dried pasta [0.1 (0.0–0.1) vs. 0.0 (0.0–0.1) g/100 g, *p* = 0.014], while a lower content in BR products was observed for sweet snacks [0.5 (0.4–0.6) vs. 0.6 (0.5–0.7) g/100 g, *p* < 0.001]. Thus, no consistency in the direction of the results was observed, with some favorable results among PL products and others among BR ones.

**Table 2:** Comparison of the nutritional quality of branded and private-label cereal-based items.

**Category**	**Brand**	**Energy** **(kcal/100 g)**	**Total fat** **(g/100 g)**	**SFA** **(g/100 g)**	**Total carbohydrates** **(g/100 g)**	**Sugars** **(g/100 g)**	**Protein** **(g/100 g)**	**Salt** **(g/100 g)**
All items	Private label	393 (354–440)	9.6 (4.1–17.0)b	1.9 (0.1–4.8)b	65.0 (52.8–71.0)	4.8 (2.1–23.0)	8.5 (7.0–11.0)	0.7 (0.4–1.4)
	Branded	395 (355–449)	10.1 (4.2–18.0)a	2.0 (0.9–5.2)a	64.5 (52.0–70.0)	4.7 (2.1–23.2)	8.6 (7.0–11.8)	0.7 (0.3–1.3)
	*p*	0.128	0.025	0.025	0.072	0.701	0.149	0.052
Breakfast cereals	Private label	384 (372–405)	3.9 (1.7–7.5)b	1.0 (0.4–3.0)b	76.0 (64.8–81.0)a	19.0 (8.5–26.5)	8.0 (7.0–9.5)b	0.5 (0.3–0.9)
	Branded	388 (372–437)	7.4 (2.9–15.0)a	1.9 (0.7–4.1)a	65.2 (57.0–75.0)b	20.4 (10.8–27.0)	8.6 (7.3–11.0)a	0.5 (0.2–0.8)
	*p*	0.119	<0.001	<0.001	<0.001	0.455	0.027	0.176
Biscuits	Private label	473 (450–487)	18.8 (16.0–21.3)	5.1 (2.3–9.3)	66.9 (63.0–70.1)	23.1 (21.0–28.0)	7.3 (6.6–8.0)	0.5 (0.3–0.7)b
	Branded	470 (449–491)	18.7 (15.5–22.4)	6.0 (2.6–11.0)	66.0 (61.9–70.0)	24.7 (20.9–29.0)	7.3 (6.4–8.1)	0.6 (0.4–0.8)a
	*p*	0.893	0.363	0.065	0.061	0.349	0.785	0.004
Sweet snacks	Private label	407 (388–425)	17.1 (15.0–21.0)b	6.5 (3.7–9.4)b	54.0 (51.0–58.0)a	28.0 (22.0–33.0)	6.5 (5.6–7.2)	0.6 (0.5–0.7)a
	Branded	408 (386–428)	19.0 (16.0–22.0)a	7.6 (4.1–10.4)a	52.0 (49.0–56.6)b	28.3 (22.0–34.9)	6.1 (5.3–7.1)	0.5 (0.4–0.6)b
	*p*	0.774	0.046	0.006	<0.001	0.501	0.062	<0.001
Bread	Private label	276 (261–286)	4.6 (3.7–5.2)	0.7 (0.5–1.0)	48.0 (45.0–51.0)	4.6 (3.3–5.8)	8.5 (8.2–9.5)	1.3 (1.2–1.4)
	Branded	271 (251–292)	4.3 (2.8–5.8)	0.7 (0.4–1.1)	47.3 (42.6–51.0)	4.4 (2.7–6.5)	8.5 (7.5–9.5)	1.3 (1.1–1.4)
	*p*	0.226	0.580	0.432	0.256	0.472	0.068	0.079
Bread substitutes	Private label	412 (383–436)	9.6 (6.8–12.8)	1.5 (0.9–2.6)	68.9 (63.0–72.8)	2.0 (1.3–3.0)	10.0 (8.2–11.1)	1.8 (1.2–2.1)
	Branded	410 (378–438)	9.6 (5.6–13.2)	1.6 (0.8–2.9)	67.5 (62.8–73.0)	2.0 (1.1–3.5)	10.0 (8.2–12.0)	1.7 (1.0–2.2)
	*p*	0.542	0.627	0.393	0.301	0.523	0.055	0.304
Fresh pasta	Private label	279 (254–293)	6.5 (3.8–8.4)b	2.6 (1.3–3.7)	41.0 (36.0–48.0)	2.2 (1.3–3.7)	11.0 (9.3–13.0)a	0.9 (0.3–1.3)b
	Branded	281 (251–298)	8.0 (5.5–10.0)a	2.9 (1.8–3.8)	39.0 (33.0–45.0)	2.5 (1.4–4.1)	9.9 (8.7–11.0)b	1.2 (0.7–1.4)a
	*p*	0.937	0.002	0.148	0.050	0.430	<0.001	0.014
Dried pasta	Private label	359 (354–365)	2.0 (1.4–3.8)b	0.5 (0.3–1.2)b	68.0 (67.0–71.5)a	2.8 (2.5–3.2)	13.0 (12.0–14.0)b	0.0 (0.0–0.1)b
	Branded	359 (351–370)	2.8 (1.7–4.2)a	0.8 (0.4–1.3)a	68.0 (66.0–71.0)b	2.8 (2.0–3.5)	14.0 (12.5–15.0)a	0.1 (0.0–0.1)a
	*P*	0.379	<0.001	0.047	0.010	0.613	<0.001	0.004

*Values are expressed as median (25th−75th percentile). Different lowercase letters indicate significant differences between PL and BR items belonging to the same category (Mann–Whitney non-parametric test for two independent samples), p < 0.05.*

*SFA, saturated fatty acids*.

These results are further confirmed by comparing the nutritional quality of PL and BR types of products within each category. Some differences indeed emerged for energy and nutrients between PL and BR products of some types in all the seven considered food categories, despite some contrasting results even within the same category.

**Figure 1:**
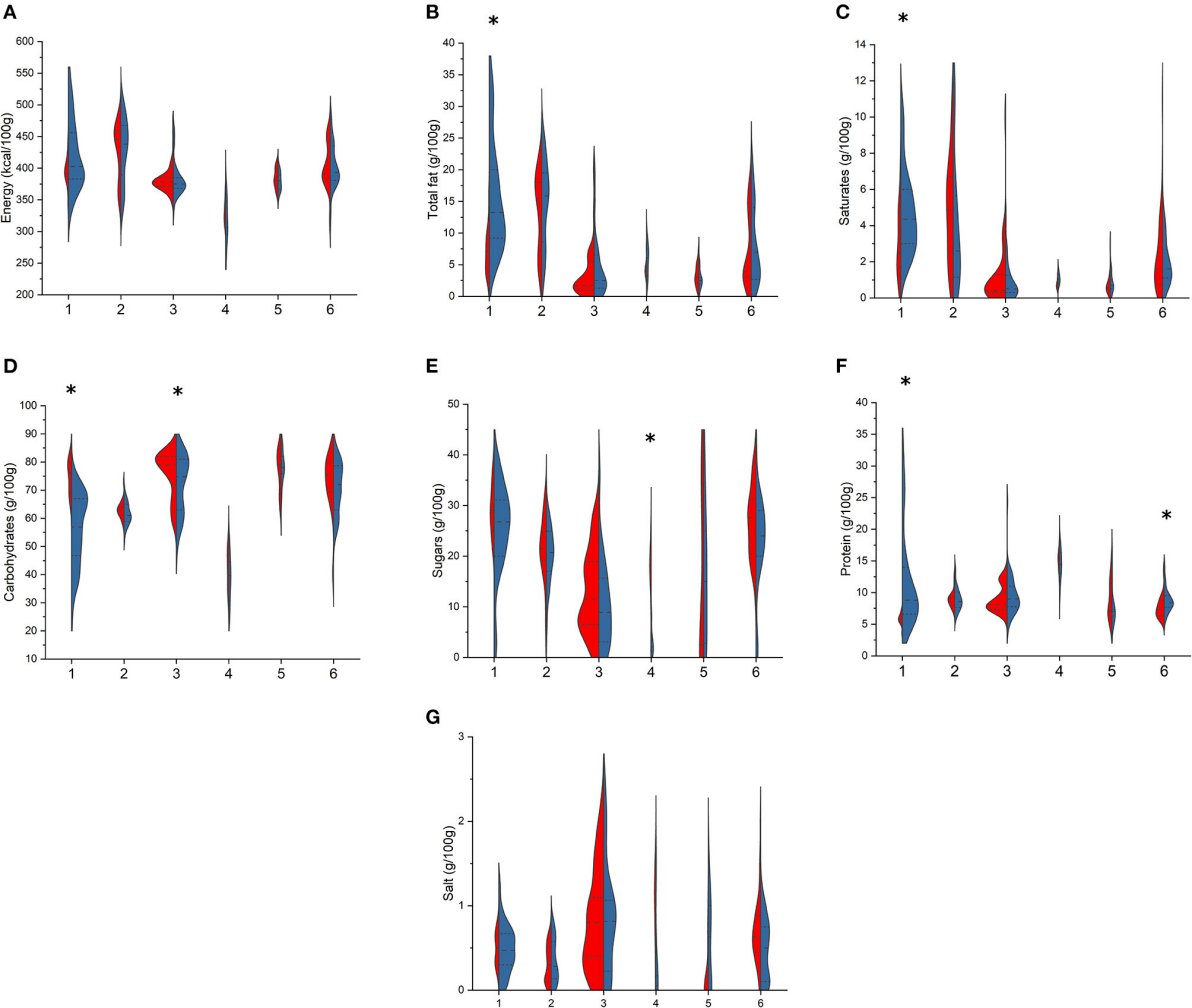
Comparison of energy **(A)**, total fat **(B)**, saturates **(C)**, total carbohydrates **(D)**, sugars **(E)**, protein **(F)**, and salt **(G)** content in branded (BR, in blue) and private-label (PL, in red) types of breakfast cereals. 1: cereal bars; 2: muesli; 3: flakes; 4: bran cereals; 5: puffed cereals; 6: other cereals. For each type, asterisk indicates significant difference between BR and PL items (Mann–Whitney non-parametric test for two independent samples), *p* < 0.05.

Among breakfast cereals (Figure 1), cereal bars showed statistically significant differences among BR and PL items with higher values in the former for total fat [13.3 (9.2–20.0) vs. 7.0 (3.8–9.0) g/100 g, *p* < 0.001], saturates [4.4 (3.0–6.0) vs. 2.5 (1.7–4.7) g/100 g, *p* < 0.009], and protein [6.0 (5.0–7.7) vs. 4.1 (2.8–5.1) g/100 g, *p* < 0.001] and lower values for total carbohydrates [56.9 (46.7–67.0) vs. 76.0 (69.0–80.0) g/100 g, *p* < 0.001]. Total carbohydrates were also lower in BR flakes compared with PL ones [74.8 (63.0–81.0) vs. 79.0 (72.0–82.0) g/100 g, *p* = 0.040], while among bran cereals, sugar content was markedly lower in BR items [2.1 (1.2–3.4) vs. 17.5 (17.0–18.0) g/100 g, *p* = 0.013, respectively], and among other cereals, protein was higher in BR compared with that in PL items [6.4 (4.0–8.1) vs. 5.1 (3.0–7.0) g/100 g, *p* = 0.016]. Energy and salt content did not differ for any of the types.

**Figure 2:**
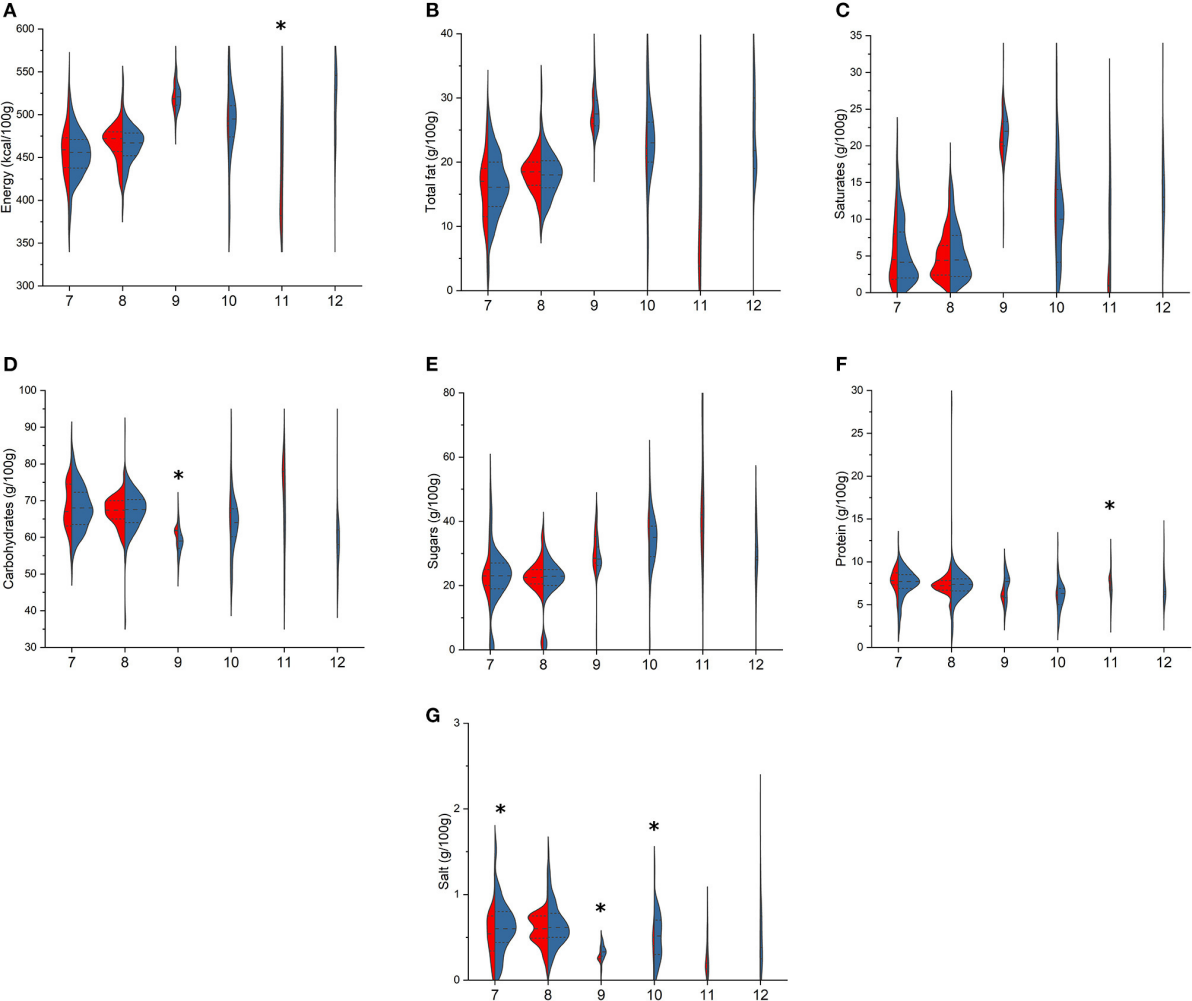
Comparison of energy **(A)**, total fat **(B)**, saturates **(C)**, total carbohydrates **(D)**, sugars **(E)**, protein **(F)**, and salt **(G)** content in branded (BR, in blue) and private-label (PL, in red) types of biscuits. 7: tea cookies; 8: shortbread biscuits; 9: cream-filled wafer; 10: covered and/or sandwich cookies; 11: Italian traditional biscuits; 12: other biscuits. For each type, asterisk indicates significant difference between BR and PL items (Mann–Whitney non-parametric test for two independent samples), *p* < 0.05.

Among biscuits (Figure 2), BR and PL traditional biscuits differed for energy [478 (433–510) vs. 423 (385–475) kcal/100 g, *p* = 0.047] and protein content [3.9 (2.6–6.0) vs. 2.0 (1.5–3.0) g/100 g, *p* = 0.029]. Compared with PL, BR wafer showed higher total carbohydrates [22.0 (20.0–23.3) vs. 20.5 (20.0–23.0) g/100 g, *p* = 0.016, respectively] and salt content [0.3 (0.3–0.4) vs. 0.2 (0.2–0.3) g/100 g, *p* < 0.001]. Higher salt content in BR than in PL items was also shown for tea cookies [0.6 (0.4–0.8) vs. 0.5 (0.3–0.8) g/100 g, *p* = 0.038] and covered/sandwich cookies [0.5 (0.3–0.7) vs. 0.4 (0.3–0.5) g/100 g, *p* = 0.042].

**Figure 3:**
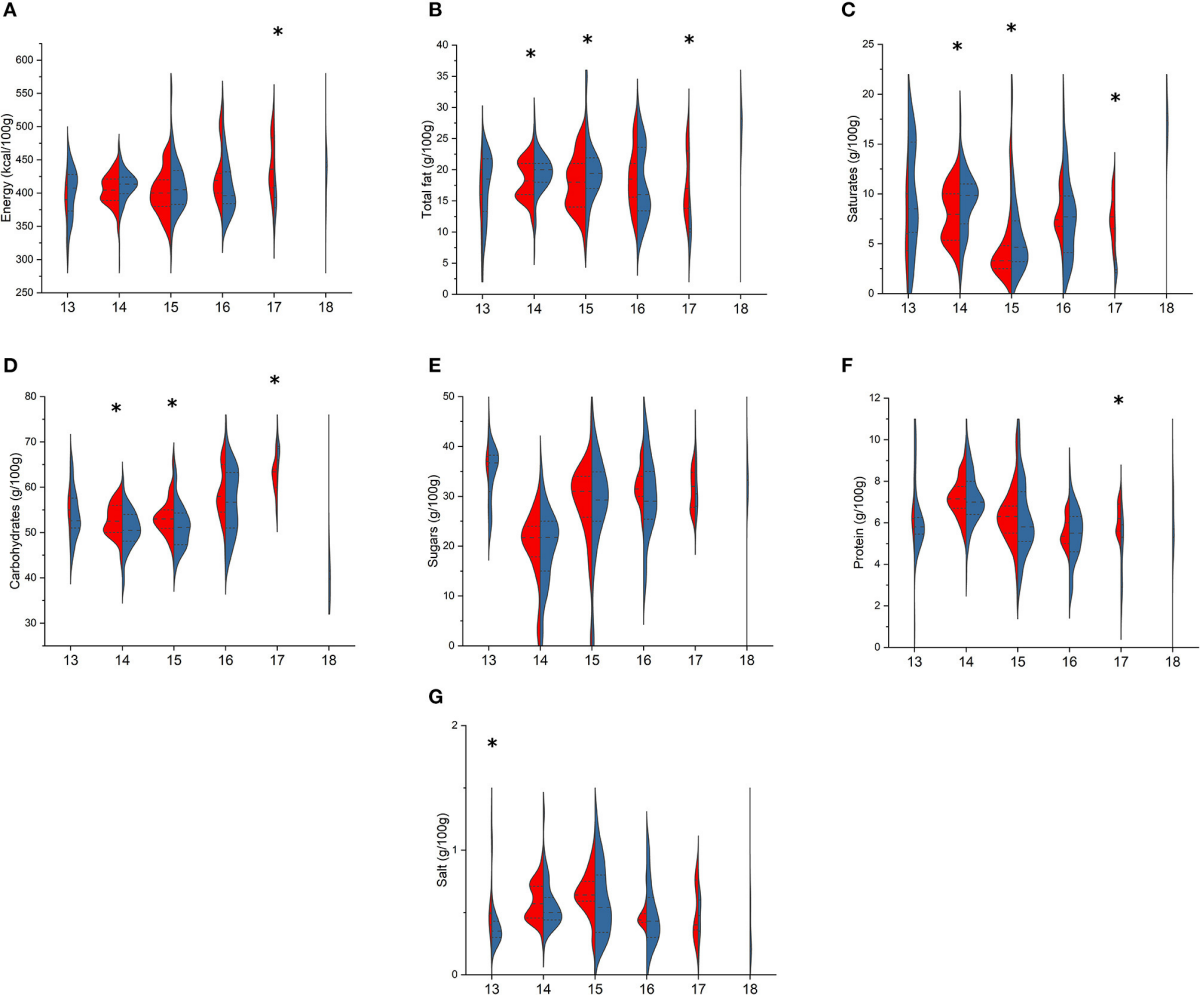
Comparison of energy **(A)**, total fat **(B)**, saturates **(C)**, total carbohydrates **(D)**, sugars **(E)**, protein **(F)**, and salt **(G)** content in branded (BR, in blue) and private-label (PL, in red) types of sweet snacks and cakes. 13: cream-filled sponge cake; 14: plain or cream/jam-filled croissant or “pain au chocolat”; 15: yogurt plumcake and muffin; 16: sponge cake; 17: cream/jam-filled shortbread cake; 18: cream-filled and/or covered chilled snack. For each type, asterisk indicates significant difference between BR and PL items (Mann–Whitney non-parametric test for two independent samples), *p* < 0.05.

Regarding sweet snacks (Figure 3), the types “plain or cream/jam-filled croissant,” “plumcake and muffin,” and “cream/jam-filled shortbread cake” differed for total fats [croissant: 20.0 (18.0–21.0) vs. 18.2 (16.0–21.0) g/100 g, *p* = 0.026; plumcake and muffin: 19.4 (17.0–21.9) vs. 18.0 (14.0–21.0) g/100 g, *p* = 0.020; shortbread cake: 10.5 (10.0–14) vs. 17.0 (13.8–23.0) g/100 g, *p* < 0.001, in BR and PL, respectively], SFA [croissant: 9.9 (7.0–11.0) vs. 8.0 (5.4–10.0) g/100 g, *p* = 0.015; plumcake and muffin: 4.7 (3.2–7.3) vs. 3.3 (2.5–4.8) g/100 g, *p* = 0.007; shortbread cake: 2.4 (2.1–3.4) vs. 7.7 (6.5–9.2) g/100 g, *p* < 0.001, in BR and PL, respectively], and total carbohydrates [croissant: 50.5 (48.1–54.0) vs. 52.5 (50.0–56.0) g/100 g, *p* = 0.007; plumcake and muffin: 51.2 (47.3–54.3) vs. 53.0 (50.9–55.0) g/100 g, *p* = 0.004; shortbread cake: 68.5 (66.0–69.0) vs. 63.4 (60.0–64.0) g/100 g, *p* < 0.001, in BR and PL, respectively]. Shortbread cake also differed for energy content [394 (378–411) vs. 436 (408–483) kcal/100 g, *p* = 0.007, in BR and PL items, respectively] (Figure 3) and for protein content, which is higher in PL than in BR items [6.2 (5.6–6.9) vs. 5.3 (2.9–5.9) g/100 g, *p* = 0.005]. Finally, cream-filled sponge cakes differed only for salt content, being higher in PL than in BR [0.5 (0.4–0.5) vs. 0.4 (0.3–0.4) g/100 g, *p* = 0.002].

**Figure 4:**
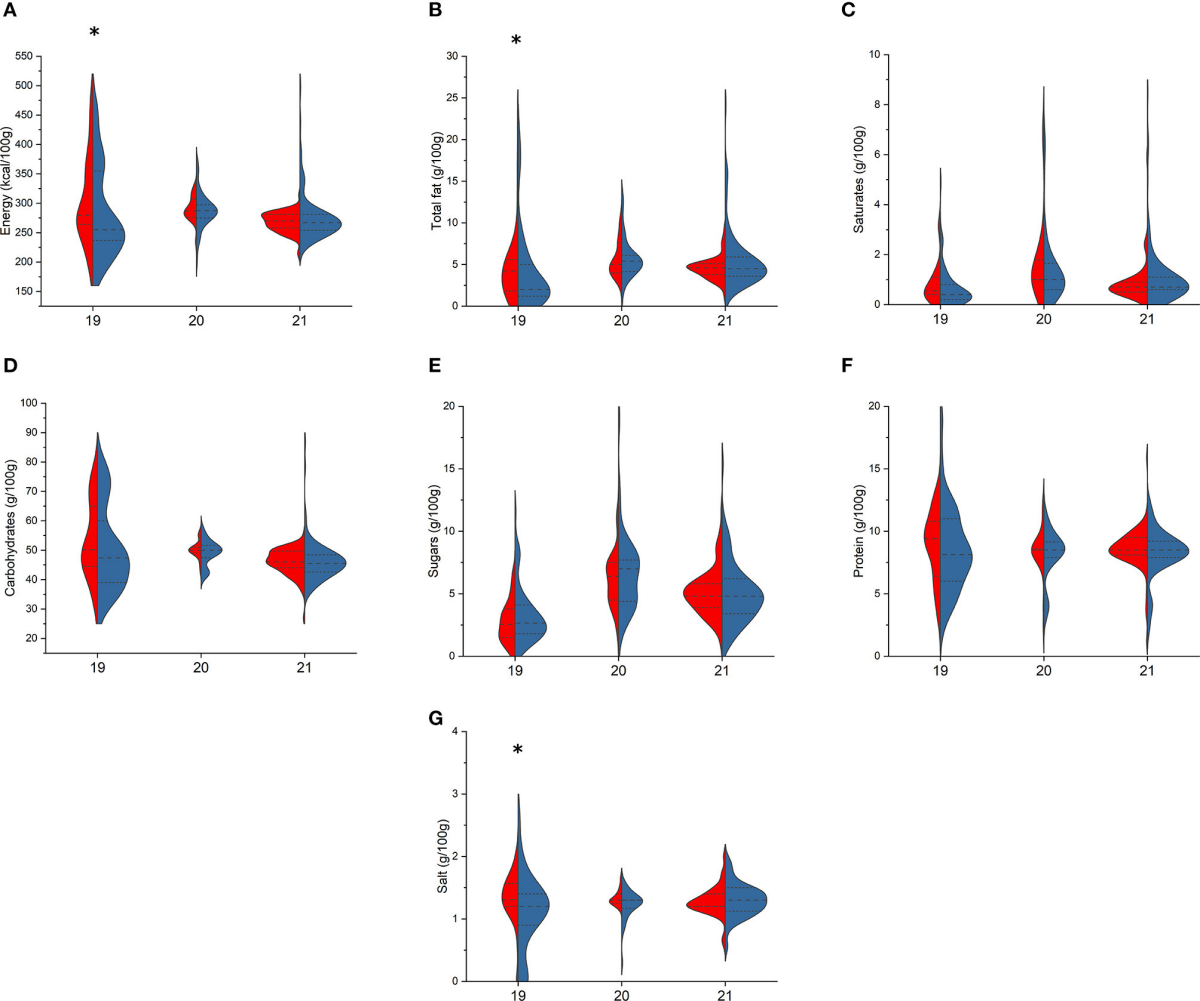
Comparison of energy **(A)**, total fat **(B)**, saturates **(C)**, total carbohydrates **(D)**, sugars **(E)**, protein **(F)**, and salt **(G)** content in branded (BR, in blue) and private-label (PL, in red) types of breads. 19: loaf bread; 20: rolls; 21: sliced bread. For each type, asterisk indicates significant difference between BR and PL items (Mann–Whitney non-parametric test for two independent samples), *p* < 0.05.

**Figure 5:**
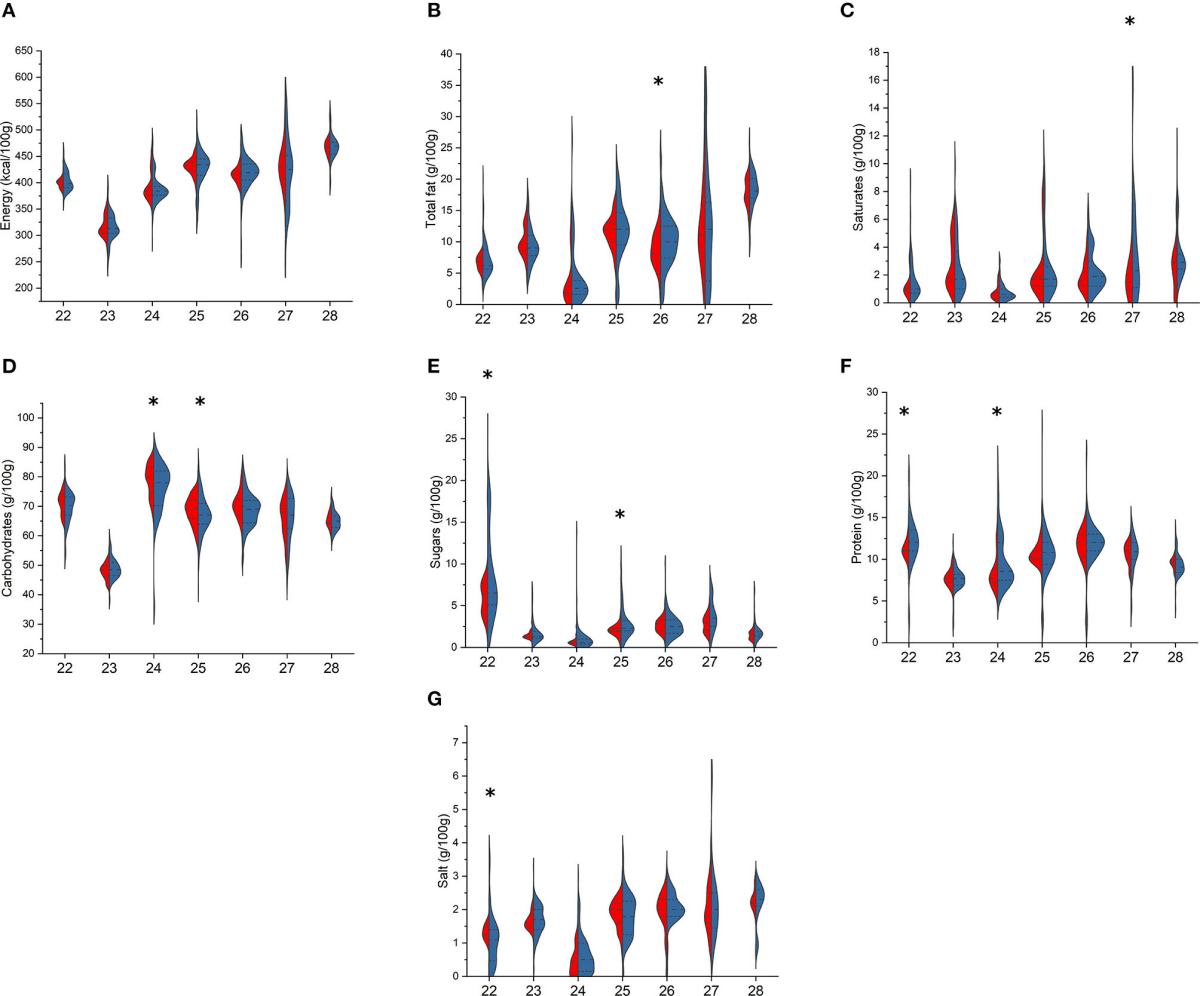
Comparison of energy **(A)**, total fat **(B)**, saturates **(C)**, total carbohydrates **(D)**, sugars **(E)**, protein **(F)**, and salt **(G)** content in branded (BR, in blue) and private-label (PL, in red) types of bread substitutes. 22: rusks; 23: wraps; 24: rice and corn cakes; 25: crackers; 26: breadsticks; 27: “croutons, bruschetta, and frisella bread”; 28: taralli. For each type, asterisk indicates significant difference between BR and PL items (Mann–Whitney non-parametric test for two independent samples), *p* < 0.05.

Comparing bread types (Figure 4), only BR and PL loaf items differed for energy [255 (237–355) vs. 280 (264–360) kcal/100 g, *p* = 0.039, respectively], total fat [2.0 (1.2–5.0) vs. 4.2 (1.8–5.6) g/100 g, *p* = 0.035], and salt contents [1.2 (0.9–1.4) vs. 1.3 (1.2–1.69) g/100 g, *p* = 0.029], while no differences were found among rolls and sliced bread. Conversely, among bread substitutes (Figure 5), BR and PL rusks differed for sugar [6.5 (5.1–11.0) vs. 6.4 (4.0–7.7) g/100 g, *p* = 0.048], protein [12.0 (11.0–13.5) vs. 11.0 (11.0–11.1) g/100 g, *p* = 0.002], and salt [1.6 (1.5–1.8) vs. 1.1 (0.5–1.4) g/100 g, *p* = 0.044] contents; breadsticks only for fat content [10.0 (7.4–12.5) vs. 8.0 (7.0–11.0) g/100 g, *p* = 0.033]; “croutons, bruschetta, and frisella bread” for saturates [2.3 (1.1–5.2) vs. 1.5 (0.9–2.5) g/100 g, *p* = 0.047]; and crackers for both total carbohydrates [67.0 (64.0–71.0) vs. 69.2 (65.9–72) g/100 g, *p* = 0.030] and sugar [2.8 (2.0–3.2) vs. 2.3 (2.0–3.2) g/100 g, *p* = 0.002]. Finally, BR “rice and corn cakes” differed to the PL ones only for total carbohydrates [78.0 (70.3–82.0) vs. 80.1 (75.0–83.1) g/100 g, *p* = 0.025] and protein [8.6 (7.5–12.0) vs. 7.7 (7.2–8.8) g/100 g, *p* = 0.010].

**Figure 6:**
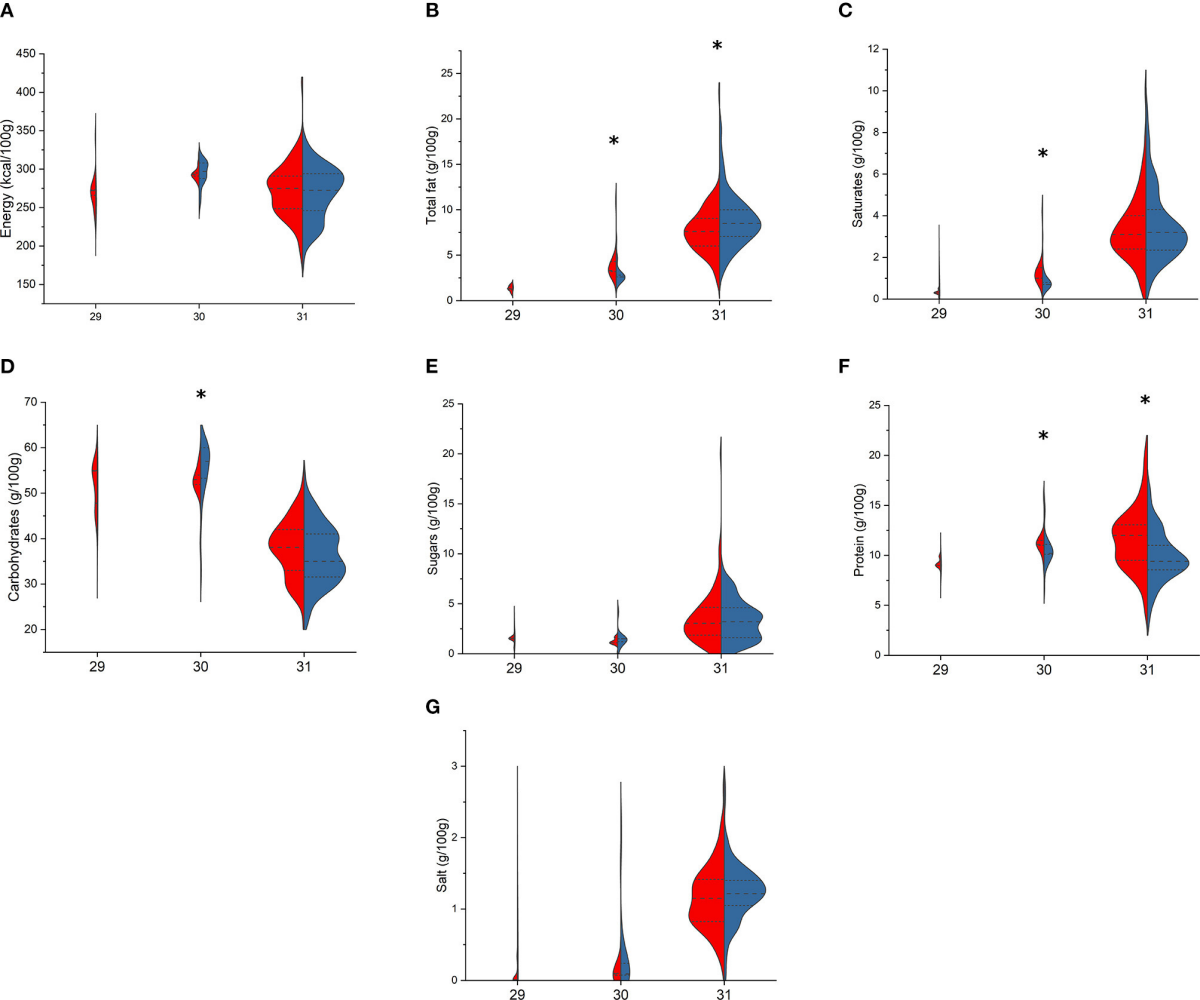
Comparison of energy **(A)**, total fat **(B)**, saturates **(C)**, total carbohydrates **(D)**, sugars **(E)**, protein **(F)**, and salt **(G)** content in branded (BR, in blue) and private-label (PL, in red) types of fresh pasta. 29: semolina pasta; 30: egg pasta; 31: stuffed pasta. For each type, asterisk indicates significant difference between BR and PL items (Mann–Whitney non-parametric test for two independent samples), *p* < 0.05.

**Figure 7:**
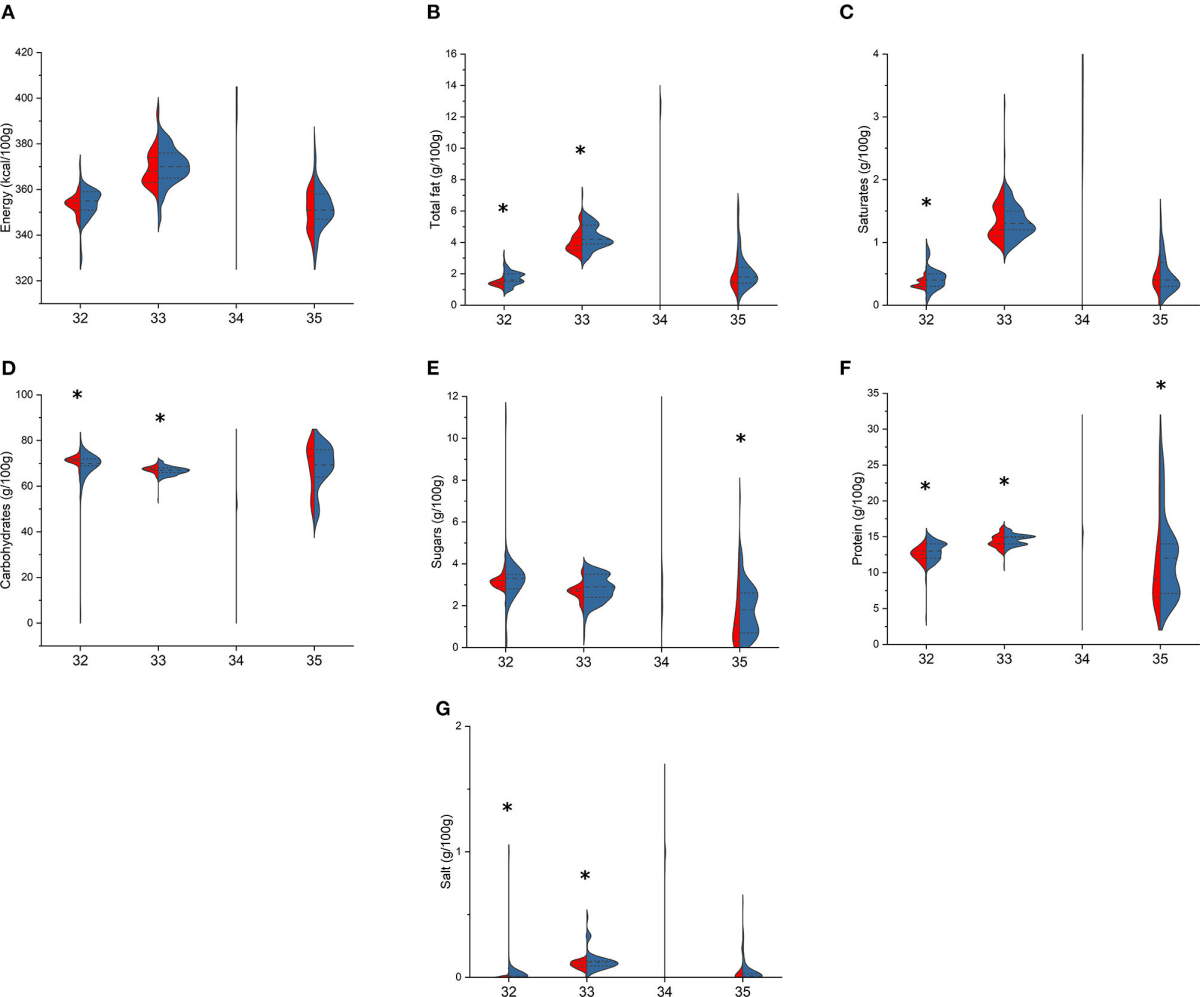
Comparison of energy **(A)**, total fat **(B)**, saturates **(C)**, total carbohydrates **(D)**, sugars **(E)**, protein **(F)** and salt **(G)** content in branded (BR, in blue) and private-label (PL, in red) types of dried pasta. 32: semolina pasta; 33: egg pasta; 34: stuffed pasta; 35: special pasta. For each type, asterisk indicates significant difference between BR and PL items (Mann–Whitney non-parametric test for two independent samples), *p* < 0.05.

Finally, considering pasta (Figures 6, 7), among fresh pasta, no differences were found between BR and PL semolina pasta. BR egg pasta compared with PL differed for lower total fat [2.6 (2.6–2.8) vs. 3.3 (3.2–4.3) g/100 g, *p* < 0.001], SFA [0.7 (0.7–0.8) vs. 1.0 (1.0–1.5) g/100 g, *p* < 0.001], and protein [10.2 (10.1–11.1) vs. 11.1 (11.0–11.5) g/100 g, *p* = 0.010] but higher total carbohydrates [57.0 (53.3–60.0) vs. 53.0 (51.9–54) g/100 g, *p* = 0.005], while BR stuffed pasta showed higher total fat [8.5 (7.1–10.0) vs. 77.6 (6.0–9.1) g/100 g, *p* = 0.002] and lower protein [9.4 (8.6–11.0) vs. 12.0 (9.5–13.1) g/100 g, *p* < 0.001] compared with the PL counterparts. Among dried pasta, BR and PL semolina and egg pasta differed for total fat, total carbohydrates, protein, and salt, while saturates differed only in semolina pasta [1.6 (1.5–2.0) vs. 1.4 (1.3–1.5) g/100 g, *p* = 0.002]. Special pasta only differed for sugar [1.8 (0.7–2.6) vs. 1.1 (0.3–2.3) g/100 g, *p* = 0.030, in BR and PL items, respectively] and protein contents [12.0 (7.1–14.0) vs. 9.0 (6.5–13.0) g/100 g, *p* = 0.033], while no differences were found between BR and PL stuffed pasta.

## Discussion

In the present study, we compared the nutrition declaration of over 3,700 cereal-based food items, belonging to seven different food categories, to investigate whether BR and PL products differ in terms of nutritional quality, by retrieving information from the food label. Overall, BR and PL products differed only for total fat and SFA contents. When considering the seven food categories, the main differences referred to the content of total fat, SFA, total carbohydrates, protein, and salt, while differences in terms of energy and sugars were not observed between BR and PL products of all categories. It is worth mentioning that these differences were often not relevant from a nutritional point of view, as the variation was generally lower than 4%. Moreover, some differences can be attributed to the ratios of BR and PL types of products belonging to the same categories. For instance, cereal bars represent ~9 and ~38% of total PL and BR breakfast cereals, respectively, while flakes ~46 and ~25%, respectively. With cereal bars and flakes very different in terms of total fat content (13), the different numbers of items in the BR and PL category might explain the higher total fat content found in BR breakfast cereals. However, we did not find any consistency in the direction of the results, with some positive profile among BR products and others among PL ones, so BR cannot be considered *tout court* as a marker of overall better nutrition quality compared with PL. In this scenario, it is noteworthy that BR did not show better nutritional values compared with PL although they bore a higher number of both nutrition and health claims compared with the related counterparts for almost all of the food categories under study.

For the abovementioned reasons and due to the heterogeneity of the characteristics of studies—in terms of considered nutrients and/or food categories—comparisons of our results with findings from previous studies are tricky. Moreover, studies were performed in different countries such as Australia (10), USA (11), and Switzerland (20), which impede a comprehensive comparison of results and do not allow the generalization of results. Regarding Australia, two studies were carried out by considering different aspects of the nutritional information on the food pack. In the first one, which considered 3,204 products (42% PL and 58% BR), total fats and SFA were significantly higher in PL than in BR products for five and seven categories, respectively, with major differences for ready meals, pastries, and salty snacks (10). Despite these categories not completely overlapping with our categories, it is worth noting that we also found significant differences for breakfast cereals and sweet snacks, but we found higher fat and SFA contents in BR than in PL items. The second Australian study compared sodium amounts in 15,680 PL and BR products from 15 food categories, finding an overall lower sodium content in PL items, as mainly found (in terms of salt) in our study for pasta—as fresh and dried—and biscuit categories (9). Another study performed in the United States analyzed the concentrations of sodium and related nutrients (potassium, total dietary fiber, total and SFA, and total sugar) on over 1,700 food items, without finding substantial differences between PL and national BR and, thus, once again suggesting that brand type is not a consideration for the nutritional quality of foods (11). This last conclusion has also been confirmed by Khalatbari-Soltani and colleagues, who compared the nutrient content of over 4,000 processed food items from 26 food categories “best price” and brand name foods in Switzerland (20). They found no differences in total energy and protein, fat, and total carbohydrates for most food categories, including breakfast cereals, cereal bars, biscuits, cakes and tarts, bread and bread products, and pasta (20). By considering the single packaged bread category, a recent Swedish study found a slightly higher nutritional quality, in terms of higher protein, lower total fat, and lower sugar, of PL items than the BR ones (21).

The study has some limitations worthy to be highlighted. First, as done for other studies performed within the FLIP project (12–17), we did not include food items from other types of retail outlets, such as discount warehouses. Secondly, the comparison of nutritional quality was based only on mandatory information based on the Regulation EU 1169/2011 (18); thus, we cannot exclude differences among other nutritional components, such as fiber, vitamins, or minerals. Moreover, it is worth mentioning that, based on the Regulation EU 1169/2011 (18), nutrition declaration can be formulated either from direct analysis of food or from data extrapolated from reference databases of food composition, which do not take into account potential differences between ingredients used in BR and PL items. Finally, it is noteworthy that the present manuscript aims to evaluate the nutritional quality of PL and BR products and to assess the overall quality of products. However, other important aspects such as the origin of the raw materials, the sensory characteristics, and many others should also be considered.

Overall, we found some contrasting results in terms of nutritional quality between BR and PL products; thus, we cannot conclude that PL items always have a lower nutritional quality than BR ones. Despite some differences among categories, this supports the theories of a net discrepancy from the perceived whole quality—nutritional, technological, and hedonistic—and effective food quality of PL foods (22), mainly driven from the perception that a branded product, higher in price, is of better quality than PL (23). Thus, as already hypothesized in the past FLIP studies (12–17), further efforts should be performed to educate consumers in reading and understanding food labels and all available information. These findings could be useful in nutrition education activities aimed to help consumers in making informed food choices and, in turn, improve their life quality. However, by considering that this study focused only on cereal-based products, future surveys focused on other food groups are needed to better elucidate possible difference in terms of nutritional declaration and ingredients among BR and PL food products currently on the Italian market.

## Data Availability Statement

The original contributions presented in the study are included in the article, further inquiries can be directed to the corresponding authors.

## Author Contributions

DA was involved in the protocol design, data analyses, and in the interpretation of results and drafted the manuscript. CD was involved in the protocol design and in the interpretation of the results and contributed to the drafting of the manuscript. NP participated in the conceptualization of the study and critically reviewed the manuscript. DM conceived and designed the protocol of the study, was involved in the interpretation of the results, critically reviewed the manuscript, and had primary responsibility for the final content. Other members of the Italian Society of Human Nutrition (SINU) Young Working Group were involved in the protocol design and critically reviewed the final manuscript. All authors contributed to the article and approved the submitted version.

## Conflict of Interest

The present publication has been conceived within the Italian Society of Human Nutrition (SINU) Young Group, and it has been made without any funding from food industries or other entities. DM has received a research grant from Despar Italia which is not related to the present study, so had no role in the design of the study; in the collection, analyses, or interpretation of data; in the writing of the manuscript; or in the decision to publish the results. The remaining authors declare that the research was conducted in the absence of any commercial or financial relationships that could be construed as a potential conflict of interest. The reviewer GS declared past co-authorship with the author NP to the handling editor.

## SINU Young Working Group

- Margherita Dall'Asta, Department of Animal Science, Food and Nutrition, Università Cattolica del Sacro Cuore, Piacenza, Italy
- Marika Dello Russo, Institute of Food Sciences, National Research Council, Avellino, Italy
- Daniele Nucci, Veneto Institute of Oncology IOV-IRCCS, Padova, Italy
- Stefania Moccia, Institute of Food Sciences, National Research Council, Avellino, Italy
- Gaetana Paolella, Department of Chemistry and Biology A. Zambelli, University of Salerno, Fisciano, Italy
- Veronica Pignone, Freelance Nutritionist, San Giorgio del Sannio, Italy
- Alice Rosi, Department of Food and Drug, University of Parma, Parma, Italy
- Emilia Ruggiero, Department of Epidemiology and Prevention, IRCCS Neuromed, Pozzilli, Italy
- Carmela Spagnuolo, Institute of Food Sciences, National Research Council, Avellino, Italy
- Giorgia Vici, School of Biosciences and Veterinary Medicine, University of Camerino, Camerino, Italy.

## References

[1] Ikonen I Sotgiu F Aydinli A Verlegh PWJ. Consumer effects of front-of-package nutrition labeling: an interdisciplinary meta-analysis. J Acad Mark Sci. (2020) 48:360–83. 10.1007/s11747-019-00663-9

[2] Calvo Porral C Levy-Mangin J-P. Food private label brands: the role of consumer trust on loyalty and purchase intention. Br Food J. (2016) 118:679–96. 10.1108/BFJ-08-2015-0299

[3] IRI. Private Label: The Journey to Growth Along Roads Less Traveled. (2016). Available online at: https://www.iriworldwide.com/IRI/media/Library/Publications/Private-Label_11-2016.pdf (accessed January 12, 2021).

[4] Nielsen Company. The Rise and Rise Again of Private Label. (2018). p. 1–21. Available online at: https://www.nielsen.com/wp-content/uploads/sites/3/2019/04/global-private-label-report.pdf (accessed January 11, 2021).

[5] Bergès-Sennou F Bontems P Réquillart V. Economics of private labels: a survey of literature. J Agric Food Ind Organ. (2004) 2:41–65. 10.2202/1542-0485.1037

[6] IRI. XVI Rapporto Marca. Bologna (2020). Available online at: http://www.marca.bolognafiere.it/marca/osservatorio-marca/xvi-rapporto-marca-by-bolognafiere/5507.html (accessed April 17, 2021).

[7] Chapman K Innes-Hughes C Goldsbury D Kelly B Bauman A Allman-Farinelli M. A comparison of the cost of generic and branded food products in Australian supermarkets. Public Health Nutr. (2013) 16:894–900. 10.1017/S1368980012000961 22475494 PMC10271420

[8] Hoch SJ Banerji S. When do private labels succeed? Sloan Manage Rev. (1993) 34:57.

[9] Trevena H Neal B Dunford E Haskelberg H Wu J. A comparison of the sodium content of supermarket private-label and branded foods in Australia. Nutrients. (2015) 7:7027–41. 10.3390/nu7085321 26308047 PMC4555160

[10] Cleanthous X Mackintosh A-M Anderson S. Comparison of reported nutrients and serve size between private label products and branded products in Australian supermarkets. Nutr Diet. (2011) 68:120–6. 10.1111/j.1747-0080.2011.01511.x

[11] Ahuja JKC Pehrsson PR Cogswell M. A comparison of concentrations of sodium and related nutrients (potassium, total dietary fiber, total and saturated fat, and total sugar) in private-label and national brands of popular, sodium-contributing, commercially packaged foods in the United State. J Acad Nutr Diet. (2017) 117:770–7.e17. 10.1016/j.jand.2016.12.001 28169210

[12] Dall'Asta M Rosi A Angelino D Pellegrini N Martini D. Evaluation of nutritional quality of biscuits and sweet snacks sold on the Italian market: the Food Labelling of Italian Products (FLIP) study. Public Health Nutr. (2020) 23:2811–8. 10.1017/S1368980020000853 32635953 PMC10200475

[13] Angelino D Rosi A Dall'Asta M Pellegrini N Martini D. Evaluation of the nutritional quality of breakfast cereals sold on the Italian Market: The Food Labelling of Italian Products (FLIP) Study. Nutrients. (2019) 11:2827. 10.3390/nu11112827 31752290 PMC6893738

[14] Dall'Asta M Angelino D Pellegrini N Martini D. The nutritional quality of organic and conventional food products sold in Italy: results from the Food Labelling of Italian Products (FLIP) Study. Nutrients. (2020) 12:1273. 10.3390/nu12051273 32365788 PMC7282013

[15] Angelino D Rosi A Ruggiero E Nucci D Paolella G Pignone V. Analysis of food labels to evaluate the nutritional quality of bread products and substitutes sold in Italy: results from the Food Labelling of Italian Products (FLIP) Study. Foods. (2020) 9:682. 10.3390/foods9050682 33419252 PMC7766686

[16] Angelino D Rosi A Ruggiero E Nucci D Paolella G Pignone V. Analysis of food labels to evaluate the nutritional quality of bread products and substitutes sold in italy: results from the Food Labelling of Italian Products (FLIP) Study. Foods. (2020) 9:1905. 10.3390/foods9121905 33419252 PMC7766686

[17] Dello Russo M Spagnuolo C Moccia S Angelino D Pellegrini N Martini D. Nutritional quality of pasta sold on the Italian market: The Food Labelling of Italian Products (FLIP) Study. Nutrients. (2021) 13:171. 10.3390/nu13010171 33429881 PMC7827935

[18] European Union. Council Regulation No 1169/2011 on the provision of food information to consumers. Off J Eur Union. (2011) L304:18–63.

[19] European Union. Council Regulation No 1924/2006 on nutrition and health claims made on foods. Off J Eur Union. (2006) L404:9–25.23712943

[20] Khalatbari-Soltani S Marques-Vidal P. Not as bad as you think: a comparison of the nutrient content of best price and brand name food products in Switzerland. Prev Med Reports. (2016) 3:222–28. 10.1016/j.pmedr.2016.02.001 27419018 PMC4929183

[21] Lappi V-M Mottas A Sundström J Neal B Löf M Rådholm K. A comparison of the nutritional qualities of supermarket's own and regular brands of bread in Sweden. Nutrients. (2020) 12:1162. 10.3390/nu12041162 32331290 PMC7230986

[22] Bao Y Bao Y Sheng S. Motivating purchase of private brands: effects of store image, product signatureness, and quality variation. J Bus Res. (2011) 64:220–6. 10.1016/j.jbusres.2010.02.007

[23] Lichtenstein DR Ridgway NM Netemeyer RG. Price perceptions and consumer shopping behavior: a field study. J Mark Res. (1993) 30:234. 10.1177/002224379303000208

