# Supplementary material for: Comparison of the Nutritional Quality of Branded and Private-Label Food Products Sold in Italy: Focus on the Cereal-Based Products Collected From the Food Labeling of Italian Products Study
Source: Front Nutr. 2021 Jun 9;8:660766. doi: 10.3389/fnut.2021.660766 (PMC8219881; doi:10.3389/fnut.2021.660766)
Supplement: Supplementary file 1 [file Table_1.docx]

**Suppl. Table 1-**Number of nutrition and health claims of private label and branded items considered in the present study.

| Category | Brand | Items with ≥1 NC | Items with ≥1 HC |
| --- | --- | --- | --- |
| *Breakfast Cereals* | Private label | 111/176 | 14/176 |
|  | Branded | 147/194 | 51/194 |
| *Biscuits* | Private label | 68/310 | 14/310 |
|  | Branded | 164/504 | 22/504 |
| *Sweet snacks* | Private label | 31/227 | 0/227 |
|  | Branded | 37/249 | 4/249 |
| *Bread* | Private label | 23/141 | 0/141 |
|  | Branded | 68/198 | 0/198 |
| *Bread substitutes* | Private label | 126/424 | 7/424 |
|  | Branded | 230/596 | 45/596 |
| *Fresh pasta* | Private label | 5/131 | 2/131 |
|  | Branded | 7/138 | 1/138 |
| *Dried pasta* | Private label | 28/173 | 6/173 |
|  | Branded | 41/314 | 29/314 |
| Total | Private label | 392/1582 | 43/1582 |
|  | Branded | 694/2193 | 152/2193 |

Data are expressed as (number of branded or private label items)/(total number of items for that category). Legend: NC: nutrition claim; HC: health claim.
